# Knowledge, attitude and practice of the Sudanese people towards COVID-19: an online survey

**DOI:** 10.1186/s12889-021-10319-5

**Published:** 2021-02-03

**Authors:** Amal Abdelrahim Osman Mohamed, Eilaf Ali Mohamed Elhassan, Abdelrahim Osman Mohamed, Awab Aldow Mohammed, Hassan Alshaikh edris, Mohamed Alhadi Mahgoop, Mohamed Emadaldin Sharif, Maab Imadeldin Bashir, Rania Bashir Abdelrahim, Wegdan Ibraheim Idriss, Elfatih Mohamed Malik

**Affiliations:** 1 Federal Ministry of Health, Khartoum, Sudan; 2 Faculty of Medicine, University of Khartoum, Khartoum, Sudan; 3 Al Nahda College, Khartoum, Sudan

**Keywords:** COVID-19, Coronavirus, Sudan, KAP study

## Abstract

**Background:**

The Novel Corona virus SARS-CoV-2 emerged to affect the human population in 2019 causing COVID-19 pandemic. The only preventive measures available are social distancing, hand washing and face masks. This study aims to assess the knowledge, attitude and practice of the Sudanese people towards COVID-19.

**Methods:**

An online cross-sectional study targeting adult Sudanese people was conducted in April 2020. The study used a self-administered questionnaire containing 18 knowledge questions, 5 questions for attitude and six questions for practices. Social media such as Facebook and WhatsApp were utilized to disseminate the questionnaire. The total number of eligible questionnaires available for analysis by the end of the period was 987.

**Results:**

The mean (±SD) age of respondents was 30.13 (±9.84) years with males representing 55.4%. The majority were university and higher education levels (95.2%), residing in Khartoum (71.7%). The mean (±SD) knowledge score of the participants was 15.33 (± 2.24) and was found to be associated with education level and age groups (*p*-value = 0.022, *P* value =0.010) respectively. The mean (±SD) attitude score was 04.15 (± 0.97) and was significantly associated with older groups and better-educated participants (p-value =0.001, p-value = 0.048) respectively. The practices related to COVID-19 preventive measures mean (±SD) was 02.58 (± 1.73) with a significant difference between age groups and area of residence.

**Conclusions:**

This study showed that the participants had good knowledge and satisfactory attitude that was not similarly expressed into practice. Efforts are needed in health education and law enforcement to improve the practices among all groups with special emphasis on younger and less educated males.

**Supplementary Information:**

The online version contains supplementary material available at 10.1186/s12889-021-10319-5.

## Background

The emergence of a new coronavirus SARS CoV-2 causing the COVID- 19 disease characterized by a wide range of symptoms and a wide range of clinical presentations (from asymptomatic to severe acute pneumonia and other multi-organ complications) represents a real threat to population worldwide [1]. The spread of the disease triggered the World Health Organization (WHO) to declare a pandemic status on March 11, 2020. Mitigation action were then followed by strong containment measures worldwide [2, 3].

The prevalence and emergence of the disease depends on infectivity and virulence of the virus itself; in addition to the interaction of the social environment, economic activities and people mobility [4]. It is clear so far that COVID-19 is rapidly spreading but the other virus aspects are not clear yet COVID- 19 virus does not have a vaccine yet, and the best way for prevention is by handwashing, social distancing, and facemask [3, 5, 6]. The first coronavirus case in Sudan was declared on March 12, 2020. Since then many cases were reported. All of these cases had recent history of travel to affected countries or close contact with a person returning from countries with reported cases. The government closed all land crossings, air and seaports and announced a public health emergency [7]. On April 18, 2020, the Sudanese government commanded total lockdown for 3 weeks in Khartoum (the capital of Sudan where most of the cases were reported) to delay the transmission of the disease. The government complained of the poor commitment of the community to the health emergency, especially during the allowed hours. To the time of writing this paper; May 6, 2020; the number of diagnosed COVID-19 cases in Sudan was 852 with 80 cases recovered and 49 deaths [8].

Sudan is the third-largest country in Africa, with approximately 60% of the population below 30 years old; Sudan has a diverse population with different cultures and religions. Besides the rich human resources, the country has many natural agricultural, animal, and mineral resources [9]. However, Sudan is also suffering from many disparities, inequalities, and civil wars that had affected various indicators.

According to the latest Multiple Indicator Cluster Survey (MICS 2014), the literacy rate among young people was 59.8% and the use of treated water was 4.1% while those who had places for handwashing were 25.8% [10]. These indicators affect how the Sudanese people respond to a pandemic such as COVID-19 that requires a high level of knowledge, and hygienic practices.

On the other hand, the country health system has been affected by the double burden of communicable and non-communicable disease with the emergence of multiple epidemics in recent years (cholera in Blue Nile state, malaria in west and chikungunya in the eastern region) [11]. All these factors plus the dramatically changing political and economic situations after the peaceful revolution made an enormous pressure on the country response to this crisis.

KAP study is a tool to understand the awareness and practices of the public towards the COVID-19 assess gaps in knowledge, practices, and preventive measures [12] the importance of this KAP study is to understand the characteristics of the community and their perception toward COVID-19, which will allow the authorities to take effective measures [13] also the control of COVID-19 depends on the community commitment rather than health regulation so recognizing the existing perception and practices may help to identify attributes that influence the public in adopting health practices and responsive behaviors [14].

This study aims to assess the knowledge, attitude, and practices of the Sudanese population towards COVID-19 to date.

## Methods

This is a descriptive cross-sectional online survey conducted through a self-administered questionnaire targeting adult Sudanese people who were living inside and outside Sudan. (The inclusion criteria were all Sudanese population, aged 18 years and more). The questionnaire was designed using Google form online questionnaire that was accessible by clicking on a link; it was disseminated by the investigators using social media such as Facebook and WhatsApp. To minimize Bias and maximize the diversity of responses the investigators distributed the questionnaire among known Sudanese Facebook pages, Whatsap groups, and advertisement Whatsap groups (paid) that have thousands of subscribers, which further disseminated the questionnaire (snowballing). The investigators followed those groups and reminded them on daily basis until the target date was reached.

The minimum sample size was calculated using the Open Epi-calculator (https://www.openepi.com/SampleSize/SSPropor.htm) to be 385, taking the margin of error as 5%, confidence interval at 95%, estimated population 40 million according to the 2008 census population projection. However, all responses during the period from April 7, 2020, to April 13, 2020 (*n* = 1013) were included to improve validity. Only 987 were included in the final analysis after exclusion of those less than 18 years, duplicates and those who did not give a consent.

### Data collection tool

The data collection tool was adapted with modifications from an online survey done in Chinese population and WHO COVID-19 rapid quantitative assessment tool [6, 15]. The questionnaire consisted of 29 items and divided into two parts (attached in the supplementary appendix). The first part investigated demographic variables and general information including age, gender, residence, and education status. The second part consisted of -multiple choice questions and yes/no questions- 4 knowledge areas with 18 questions (mode of transmission, symptoms, at-risk groups, preventive measures); 5 attitude questions (the danger of the disease, handwashing, masks use, social distancing and isolation of diseased patients) and 3 practices areas (handwashing, social distancing and handshaking) including questions about family practice regarding the use of mask and social distancing.

### Data management

The knowledge, attitude and practices scores were calculated as one for yes answer and zero for no answer. Then each item was calculated separately. The mean and standard deviation for the knowledge attitude and practices scores were obtained. In the practices the Yes frequently answer were considered as yes while yes sometimes and no were considered as no. .

### Data analysis

Data was analyzed using IBM SPSS version 25. Frequencies were described for all independent and dependent variables. Then analysis of variance using a t-test and F test to determine the association between knowledge, attitude and practice and the independent variables of age, gender, and residence and education status was done. Binary logistic regression was used to determine factors associated with knowledge and practices scores (the good knowledge and practices were considered as scores above the mean). The statistical significance level was set at *p* < 0.05.

## Results

The mean (±SD) age of the study sample was 30.13 years (± 9.84). Males represent 55.6% (*n* = 549) of the subjects. The majority of the respondent’s education level was university and more (95.2%) and most of them resided in Khartoum state (Table 1). The awareness about COVID-19 pandemic was found to be 91.3% among the study population; the major source of information was official websites of the Ministry of Health (MOH) and WHO (Table 1).

**Table 1:** Sociodemographic characteristics of the participants (*n* = 987)

Variable	Frequency	Percentage (%)
**Age groups in years**		
Less than 30 years	576	58.4
30 years and more	411	41.6
**Mean (±SD) age in years**	30.13 (± 9.84)	
**Gender**		
Female	438	44.4
Male	549	55.6
**Highest education level**		
Basic education	47	4.8
University and more	940	95.2
**Residence**		
Khartoum State	708	71.7
Other states	168	17.0
Sudanese but currently outside Sudan	111	11.2
**Awareness about COVID-19 pandemic**		
Yes	901	91.3
No or not sure	86	8.7
**Source of knowledge (n for each is 987)**		
Official websites (e.g. MOH, WHO)	775	78.5
Social media	471	47.7
Mass media	463	46.9
Public health/ hospitals banners	411	41.6
Health personnel	244	24.7
Social talks (friends, family and co-workers)	213	21.6

The mean knowledge score was found to be 15.33 (± 2.24) with the older group (30+ years) having a significantly higher mean of knowledge (*P*-value = 0.010). There was a significant difference between the education groups in the means of knowledge scores towards COVID-19 (*p*-value =0.022) (Tables 2 and 3).

**Table 2:** Knowledge, attitude and practice of COVID-19 among Sudanese

Variable	Frequency (%)
**Knowledge**	
A. **COVID-19 can be transmission through**	
1. Airborne	**276 (28.0)**
2. Droplets	881 (89.3)
3. Fomite and surfaces	878 (89.0)
B. **Symptoms of COVID-19 include**	
4. Cough	846 (85.7)
5. Fever	916 (92.8)
6. Headache	865 (87.6)
7. Tiredness	736 (74.6)
8. Difficulty in breathing	943 (95.5)
9. Sore throat	839 (85.0)
C. **Risk of developing severe form of COVID-19 include**	
10. Chronic diseases	789 (79.9)
11. Pregnancy	289 (29.3)
12. Old age	806 (81.7)
13. Any one (irrespective to his health condition or age)	356 (36.1)
14. Do not know any risk	025 (02.5)
D. **Preventive measures for COVID-19 include**	
15. Hand washing	969 (98.2)
16. Social distancing	933 (94.5)
17. Wearing a mask	760 (77.0)
18. Antibiotics	046 (04.7)
19. Specific medicine for treatment of COVID −19	029 (02.9)
**Attitude**	
1. Hand washing is important in controlling the spread of COVID-19	912 (92.4)
2. Wearing masks is important in controlling the spread of COVID-19	491 (49.7)
3. Isolation of suspected would prevent the spread of the virus	918 (93.0)
4. Social distancing would prevent the spread of the virus	889 (90.1)
5. COVID-19 seems a dangerous disease	883 (89.5)
**Practice**	
1. I am practicing social distancing	601 (60.9)
2. I am practicing hand washing frequently	684 (69.3)
3. I avoid shaking hands	266 (27.0)
4. My family practiced social distancing frequently	293 (29.7)
5. My family practiced hand washing frequently	567 (57.4)
6. My family used face masks frequently	133 (13.5)
**Overall**	
Overall Knowledge score (Mean ± SD)	15.33 (± 2.24)
Overall attitude score (Mean ± SD)	04.15 (± 0.97)
Overall practices score (Mean ± SD)	02.58 (± 1.73)

**Table 3:** Predictors of Sudanese knowledge, attitude and practices related to COVID-19

Factor variables	Knowledge			Attitude			Practice		
	Mean (SD)	F/t-test	*P*-value	Mean (SD)	F/t-test	*P*-value	Mean (SD)	F/t-test	*P*-value
**Age groups in years**									
Less than 30 years	15.17 (2.37)	2.587	0.010	4.12 (0.97)	0.969	0.001	2.33 (1.69)	29.537	< 0.001
30 years and more	15.55 (2.04)			4.18 (0.97)			2.93 (1.73)		
**Gender**									
Female	15.35 (2.29)	0.307	0.759	4.20 (0.91)	1.623	0.105	2.66 (1.69)	1.589	0.208
Male	15.31 (2.21)			4.10 (1.02)			2.52 (1.76)		
**Highest education level**									
Basic education	14.60 (2.53)	2.301	0.022	3.87 (1.21)	1.983	0.048	2.19 (1.80)	2.457	0.117
University and more	15.37 (2.22)			4.16 (0.96)			2.60 (1.73)		
**Residence**									
Khartoum State	15.38 (2.26)	0.643	0.526	4.14 (0.94)	1.534	0.216	2.52 (1.68)	50.269	< 0.001
Other states	15.16 (2.41)			4.07 (1.15)			1.92 (1.68)		
Sudanese currently outside	15.29 (1.85)			4.28 (0.86)			3.92 (1.42)		

The overall attitude score of the participants was 04.15 (± 0.97); and there was significant difference between the age groups (P-value = 0.001) as well as different levels of education (*P*-value = 0.048). On the other hand, the mean practices score was 02.58 (± 1.73), with a significant difference between age groups and area of residence (Tables 2 and 3).

Analysis of practice items have shown that: hand washing and avoidance of handshaking were significantly associated with age and area of residence (*P*-value < 0.001); the data also showed that female practiced hand washing more frequently than males (*P*-value = 0.045) (Table 4).

**Table 4:** predictors of practices items related to COVID-19

Variable	I practice hand washing frequently			I practice social distancing		I avoid hand shaking			
	mean	F/t test	***P*** value	mean	F/t test	***P*** value	mean	F/t test	***P*** value
**Age groups**									
**Less than 30 years**	**0.62**	**37.854**	**< 0.001**	**0.60**	**01.047**	**0.307**	**0.23**	**13.644**	**< 0.001**
**30 and more**	**0.80**			**0.63**			**0.33**		
**Gender**									
Female	**0.73**	**04.044**	**0.045**	**0.64**	**02.607**	**0.107**	**0.29**	**01.672**	**0.196**
Male	**0.67**			**0.59**			**0.25**		
**Highest education level**									
Basic education	**0.57**	**3.264**	**0.071**	**0.57**	**0.245**	**0.620**	**0.23**	**0.315**	**0.575**
University and more	**0.70**			**0.61**			**0.27**		
**Residence**									
Khartoum State	**0.70**	**23.693**	**< 0.001**	**0.60**	**19.767**	**< 0.001**	**0.23**	**73.734**	**< 0.001**
Other states	**0.52**			**0.49**			**0.15**		
Sudanese currently outside	**0.90**			**0.86**			**0.71**		

Further analysis of the data using binary logistic regression (Tables 5, 6) displayed that better knowledge was associated with older age and higher education. Moreover, better practices were associated with older age and female gender. There was no significant association between the practices and the knowledge and attitude.

**Table 5:** Results of binary logistic regression on factors associated with knowledge score

Variable	OR (95% CI)	***P*** value
**Age groups(less than 30 vs 30 and more)**	**0.618 (0.470–0.812)**	**0.001**
**Gender male (vs female)**	**1.224 (0.939–1.596)**	**0.135**
**Education university and more (vs no university)**	**0.519 (0.283–0.953)**	**0.034**
**Area of residence**		**0.195**
**Area of residence Khartoum state (vs others)**	**1.419 (0.927–2.172)**	**0.107**
**Area of residence outside Sudan(vs others)**	**1.213 (0.859–1.712)**	**0.273**

**Table 6:** Results of binary logistic regression on factors associated with practices score

Variable	OR (95% CI)	***P*** value
**Age groups(less than 30 vs 30 and more)**	**2.022 (1.531–2.670)**	**< 0.001**
**Gender male (vs female)**	**1.537 (1.170–2.020)**	**0.002**
**Education university and more (vs no university)**	**0.816 (0.428–1.554)**	**0.535**
**Area of residence**		**< 0.001**
**Area of residence Khartoum state (vs others)**	**4.460 (2.614–7.611)**	**< 0.001**
**Area of residence outside Sudan(vs others)**	**0.584 (0.408–0.838)**	**0.003**
**knowledge score good (vs poor)**	**1.152 (0.883–1.502)**	**0.296**
**Attitude score good (vs poor)**	**1.050 (0.805–1.370)**	**0.719**

## Discussion

This study was performed in a time of a quickly changing evidence and rapid spread of the coronavirus. The study was performed among the Sudanese population using smartphones and social media that explain why the majority of the population were young, living in Khartoum state and were university or highly educated. This was also the case in previous t quick online surveys on COVID-19 [6, 14, 16–18].

The high awareness in this study can be explained by the explosive flow of information on all platforms especially after the appearance of the first case of the disease in the country similar to what happened in China [6]. The sources of knowledge were mainly official websites (internet) and social media. The Sudanese Federal Ministry of Health has a well-established with thousands of flowers, continuously updated social media page and an official website. The wide access to the social media carries the risk of infodemics which the authorities should mitigate by proper risk communication [17, 18]. Similar to what happened in Egypt, hydroxychloroquine ran out of the stocks in Sudan after rumors of its promising effect on COVID-19 [17].

The knowledge of the population was high and this may be attributed to the characteristics of the study population, it is also a feature found in other countries in the region [17–20]. However, another multinational study indicated lower knowledge in countries in the region (Jordan, Kuwait and Saudi Arabia) [21]. The data revealed that well-educated and older persons had better knowledge about the COVI-19; this can be used to direct the health education efforts towards younger and less educated groups.

It’s worth noting that 98, 95 and 77% of the population had knowledge about the main preventive measures of the disease spread (handwashing, social distancing, and masks) if this knowledge has been transferred into practice it would make significant difference in the control of the disease.

The attitude of the participants was generally good, with positive significant difference among older and educated groups this was consistent with their knowledge and is consistent with other studies [6, 14].

The attitude about wearing face masks was low (49%) compared to 77% of the population mentioned it as a mean for controlling the disease. This could be attributed to several factors: inconsistency in the information between different sources such as WHO, CDC, and MoH which was the same case in Egypt [16, 17]. There was no law enforcement of the masks use unlike the situation in China and Saudi Arabia [6, 14]. Other possible factors include the high prices of masks and their availability in the Sudanese market, the latter factor has been a global issue [16].

The mean practices score was relatively low compared to high knowledge and good attitude of the participants. This finding brings the importance of improving the accessibility to preventive measures such as availability of handwashing facilities, facemasks, and enforcing social distancing in different facilities.

The Sudanese community is a sociable active community which influenced the avoidance handshaking (27%) and social distancing unlike the situation in other countries like Saudi Arabia where 88% avoided handshaking [14].

This study demonstrated that the area of residence affects the practices related to the preventive measures especially in those outside Sudan despite the lack of the significant effect on the knowledge and attitude; this might be explained by accessibility to hygiene materials and strict regulations.

It is known that hand hygiene is a major element in the prevention of COVID-19 and other infectious disease. Poor hand washing practice is more linked with male gender, younger age and residence outside Khartoum. This might be due to several factors such as males and young people tend to take more risky behavior as shown in different studies [6]. Beside that in the states, the social networks are more active with less available services.

The good knowledge and attitude has not been translated to satisfactory practices. Which will affect the combating of this virus as the action is mainly based on the community engagement and behavioral changes [14].

### Implication of the findings on the health system

The weak infrastructure, under-resourced health system, widespread of the illiteracy and social practices will negatively influence the spread of the COVID-19 and response towards its prevention.

The study population may not represent the diverse Sudanese communities but it gives an insight to what is happening in Sudan. It is expected that the situation to be worse in more remote and underprivileged areas.

### Limitations

The study was conducted while the country started adopting restrictive measures thus investigators were forced to use online survey methods with its limitations. Access to smartphones and internet is known to be in high and middle socioeconomic classes and among younger age groups and urban areas. The study population may not represent the diverse Sudanese people and the results of this study should not be generalized. Another limitation is the inadequate assessment of the attitudes towards the COVID-19 as the attitude should be measured using Likert scales with more series of questions.

## Conclusions

This study showed that the participants had good knowledge and satisfactory attitude that was not similarly expressed into practice. Efforts are needed in health education and law enforcement to improve the practices among all groups with special emphasis on younger and less educated males.

## Supplementary Information

**Additional file 1.**

## Data Availability

All data generated or analyzed during this study are included in this published article and its supplementary information files.
